# Dietary forage to concentrate ratios impact on yak ruminal microbiota and metabolites

**DOI:** 10.3389/fmicb.2022.964564

**Published:** 2022-08-11

**Authors:** Kaiyue Pang, Shatuo Chai, Yingkui Yang, Xun Wang, Shujie Liu, ShuXiang Wang

**Affiliations:** ^1^Qinghai Academy of Animal Husbandry and Veterinary Sciences in Qinghai University, Xining, Qinghai, China; ^2^Key Laboratory of Plateau Grazing Animal Nutrition and Feed Science of Qinghai Province, Xining, Qinghai, China; ^3^Yak Engineering Technology Research Center of Qinghai Province, Xining, Qinghai, China

**Keywords:** yaks, forage to concentrate ratio, rumen, microbiota, metabolomics

## Abstract

To improve the rumen fermentation function and growth performance of yaks (Bos grunniens), better understanding of the effect of different dietary forage to concentrate ratios on rumen microbiota and metabolites is needed. In the present study, three diets with different dietary forage to concentrate ratios (50:50, 65:35, and 80:20) were fed to 36 housed male yaks. The changes in the distribution of rumen microorganisms and metabolites and the interactions between them were studied by 16S rRNA gene sequencing and liquid chromatography-mass spectrometry (LC–MS). The diversity and richness of microorganisms in the rumen varied according to diet. The most abundant phyla were Firmicutes and Bacteroidetes. Firmicutes was the most abundant in the C50 group, and the relative abundance of Bacteroidetes was significantly lower in the C65 group than in the C80 group (*p* < 0.05). The Christensenellaceae_R-7_group, Rikenellaceae_RC9_gut_group, and Methanobrevibacter had the highest relative abundances at the genus level. Among them, Christensenellace_R-7_group had the highest relative abundance in the C50 group. The Rikenellaceae_RC9_gut_group was significantly abundant in the C80 group compared with the C50 group. The Methanobrevibacter content was higher in the C65 group than in the other two groups. Both the concentration and metabolic pathways of rumen metabolites were influenced by the dietary concentrate ratio; lipids, lipid-like molecules, organic acid metabolites, and organic oxide-related metabolites differed between the groups. Significant changes were found for six metabolic pathways, including arginine and proline metabolism; glycine, serine, and threonine metabolism; glyoxylate and dicarboxylate metabolism; arginine biosynthesis; glycerophospholipid metabolism; glycerolipid metabolism; and nitrogen metabolism.

## Introduction

Yaks (*Bos grunniens*), an endemic livestock species living on the Qinghai–Tibetan Plateau (QTP), are an important means of production and livelihood for local herders (Long et al., 2008). In recent years, with the improvement of human living standards, human demand for livestock products is increasing. To meet the increasing demand for livestock products in the market, the production performance of ruminants is generally improved by increasing the proportion of concentrate feed in the diet. However, in actual production, achieving the optimal proportion of concentrate feed in the diet is rare; concentrations that are too high or too low affect the economic efficiency of breeding. Therefore, it is important to choose an appropriate ratio of dietary forage to concentrate to improve the breeding of yak.

The rumen is an important site for the digestion of feed in ruminants and contains a large number of bacteria, fungi, protozoa, and archaea (Qiu et al., 2020). Ration composition and nutrient content are the most important factors influencing rumen microbiota (Yáñez-Ruiz et al., 2015). Therefore, understanding the response of the rumen microbiota of ruminants to dietary changes is essential to improve the growth performance of ruminants. The effect of diet structure on the rumen microbiota of yaks has become a new hot topic in recent years. At present, many studies have investigated the rumen microbes of yaks (Fan et al., 2021; Wu et al., 2021). For example, Ahmad et al. (2020) investigated the effect of different dietary energy levels on the rumen microbiota of yaks. However, there is little published information on the effect of dietary concentrate to forage ratio on the composition of the yak rumen microbial community, therefore, our study focused on this area.

Since the health and growth of ruminants depend heavily on the production of metabolites in the rumen, a comprehensive analysis of rumen fluid provides insight into the interaction between the diet fed to ruminants and the rumen (Saleem et al., 2013). In recent years advanced techniques have been able to identify the metabolites of rumen microbiota, and it has been found that changes in some metabolites may affect microbiota alterations and host activity. Previous studies have shown that approximately 55%–60% of rumen fluid metabolites are associated with rumen microbiota (Saleem et al., 2013). The interaction between microbiota and metabolites facilitates the interpretation of microbiota classification and functional properties of some metabolites (Heinken et al., 2014). Studies on rumen microbiota and metabolites in yaks have revealed that the microbiota composition is significantly different between the concentrate and roughage groups and that the type of feed significantly alters the concentration and metabolism of metabolites involved in protein digestion and absorption (Liu et al., 2019b). Studies on the rumen microbiota and metabolic profile of Holstein heifers found that rumen microbiota were significantly affected and rumen metabolites amino acids, lipids, organic acids, and carbohydrates were significantly changed by changing the dietary forage to concentrate ratios (Zhang et al., 2017).

Although microbiomic and metabolomic studies have been widely used in animal nutrition, there is a lack of reports on the interaction between rumen microbiota and metabolites in yaks at different dietary forage to concentrate ratios, mainly owing to the differences in nutritional and metabolic characteristics between yaks and other breeds of cattle, the specificity of the growing environment, and the lack of technical guidance. Therefore, it is possible that the results of other ruminant studies may not be adapted to the diets of yaks. In this study, the rumen microbiota and metabolites of yaks were analyzed by combining 16S rRNA gene sequencing and LC–MS techniques to illustrate the effects of three different dietary forage to concentrate ratios.

## Materials and methods

### Animals, diets, and experimental design

This study was conducted from September to December 2019 at the Laozaxi breeding site in Guinan County, Qinghai Province, China. In total, 36 3-year-old healthy male yaks (weight: 164.9 ± 12.9 kg) were selected from grazing pastures and randomly divided into three groups. The yaks in the three groups were fed diets with a 50:50 (C50), 65:35 (C65), or 80:20 (C80) concentrate to forage ratio. According to the survey, the current level of concentrate used in farms is about 50–80%, however, the level of concentrate of high concentrate is usually 70 -80%, and the level of concentrate below 50% does not have the effect of promoting production, so we screened between 50 and 80% and set a three gradient concentrate to forage ration. The diets were prepared according to the Chinese Beef Cattle Feeding Standard (NY/T815-2004), and the composition and nutrient content of the diets are shown in Table 1. All yaks were uniformly numbered and fed alone with free access to water and fed twice a day at 08:00 and 17:00. The pretest period was 15 days, and the normal test period was 90 days.

**Table 1:** Ingredients and nutritional composition of each diet.

Ingredients (%)	Group		
	C50	C65	C80
Oat hay	50.00	35.00	20.00
Corn	22.68	29.75	37.15
Wheat	6.25	8.39	10.42
Wheat bran	6.43	8.56	10.67
Rapeseed meal	6.36	8.55	10.61
Soybean meal	2.17	2.91	3.62
Palm oil powder^2^	2.11	2.84	3.53
CaHPO4	1.00	1.00	1.00
NaCI	1.00	1.00	1.00
Premix^1^	2.00	2.00	2.00
**Nutrient composition (%)**			
CP	12.41	13.18	13.87
ME MJ/kg	10.98	11.89	12.80
NDF	37.67	31.36	25.48
ADF	23.32	18.36	13.61
Ca	0.45	0.48	0.50
P	0.48	0.56	0.63

1 The premix provided the following per kg of the diet: VA 3500 IU, VD 1000 IU, VE 40 IU, Mn 40 mg, Fe 50 mg, Cu 10 mg, Zn 40 mg, Se 0.3 mg;

2 Others were measured values.

Crude protein (CP), neutral detergent fiber (NDF), acid detergent fiber (ADF), calcium (Ca), and phosphorus (P) content were determined according to the official reference method were determined in each sample in the laboratory, and ME was calculated. Mixed feeds (100 g) were collected and dried in a forced-air oven at 60°C for 48 h and then ground through a 1-mm sieve before analysis. CP、Ca and N contents were determined according to AOAC (2007). The NDF and ADF contents were determined by the method of Vansoest et al. (1991).

### Rumen sample collection and measurements

On day 90 of the formal trial, 150 ml of rumen fluid samples were collected using a gastric tube sampler before morning feeding. Ruminal fluid was collected from 36 yaks, and five ruminal fluid samples were randomly selected from each group for measurement and gene sequencing. Rumen fluid samples were filtered through 4 layers of gauze and rumen pH was measured immediately using a benchtop acidity meter (Model HI221, HANNA, Italy). The remaining rumen fluid samples were dispensed into 15 ml centrifuge tubes and immediately frozen in liquid nitrogen, then transferred to a − 80°C refrigerator. The filtered rumen fluid was centrifuged (17,000 × *g* for 30 min at 4°C) to obtain the supernatant, which was further analyzed for NH_3_-N using phenol hypochlorite analysis (Broderick and Kang, 1980). Freshly prepared metaphosphoric acid (25% w/v, 2 ml) was added to 8 ml of filtered rumen fluid and then centrifuged (17,000 × *g* for 10 min at 4°C). Volatile Fatty Acids (VFAs) concentrations were determined using gas chromatography (GC-2014; Shimadzu Corporation, Japan) as described by Cao et al. (2008). The determination conditions were as follows: flame ionization detector (FID), capillary column (FFAP, 30.00 m × 0.32 mm × 0.50 μm); The heating conditions were as follows: the initial temperature was 60°C, and the temperature was increased to 120°C at 10°C/min and retained for 2 min; the temperature was increased to 180°C at 15°C/min and retained for 5 min; the vaporization chamber temperature was 250°C. FID temperature 250°C; Sample size: 1 μl, carrier gas: high purity nitrogen (99.99%), pressure: 0.7 MPa; Hydrogen pressure 0.4 MPa, air pressure 0.4 MPa, capillary column pressure 0.6–0.8 MPa, shunt ratio 40:1.

#### Microbial DNA extraction, PCR amplification, sequencing, and sequencing data processing

The CTAB method was used to extract microbial DNA from rumen fluid samples. We used 1.0% agarose gel electrophoresis to detect the concentration and purity of DNA. According to the concentration, the DNA was diluted to 1 ng/μl with sterile water. The extracted DNA was amplified by PCR, using barcoded specific primers 515F (5′-GTGCCAGCMGCCGCGG-3′) and 806R (5′-GTGCCAGCMGCCGCGG-3′) to amplify the V3 – V4 hypervariable region of 16S rRNA gene. We used 25 μl amplification system, 5 μmol/l upstream and downstream primers, and ~5 ng template DNA for the PCR reaction. PCR amplification conditions were as follows: 94°C pre-denaturation treatment for 5 min, a denaturation cycle at 94°C for 30 s, annealing at 50°C for 30 s, and extension at 72°C for 60 s for a total of 30 cycles; finally, a 72°C extend for 7 min. The PCR amplification products were detected by 1.0% agarose gel electrophoresis; the recovered products were purified using the MinElute Gel Extraction Kit (Qiagen, Germany) and TruSeq® DNA PCR-Free Sample Preparation Kit (Illumina, United States). The constructed library was quantified by Qubit and Q-PCR, and then sequenced on the Illumina NovaSeq PE250 platform. PCR amplification, PCR product mixing and purification, library construction, and computer sequencing processes were all produced by Novogene Bioinformatics Technology Co., Ltd (Beijing, China) to produce 250 bp paired-end readings.

The sequences obtained from the NovaSeq PE250 platform were processed through open-source software pipeline QIIME (Quantitative Insights into Microbial Ecology) version 1.8.0-dev (Caporaso et al., 2010), with the criteria as described by previous reports (Mao et al., 2015; Ye et al., 2016). Briefly (1) threads that had a mean quality score of no <20 and no shorter than 50 bp were retained; (2) we discarded reads that had exact barcode matching, two nucleotide mismatches in primer matching, and/or ambiguous characters; (3) only sequences that overlapped by more than 10 bp were assembled according to their overlap sequence. Reads that could not be assembled were discarded. Sequences were binned into operational taxonomic units (OTUs) based on 97% identity using UCLUST (version7.1,^1^ and chimeric sequences were identified and removed by UCHIME (Edgar, 2010). The most abundant sequence within each OTU from specific libraries (bacteria) was designated as the “representative sequence” and aligned against the (SILVAbacterial) database (version 119; Pruesse et al., 2007), with the default parameters set by QIIME. Community richness and diversity, analyzed with measures such as Good’s coverages, observed species, PD whole tree, Chao1 and Shannon indices, weighted uniFrac distance-based principal coordinate analysis (PCoA), and weighted distance-based analysis of molecular variance (AMOVA), which were used to illustrate significant differences among the samples, were assessed by the program MOTHUR v.1.35.0 (Schloss et al., 2009).

All the raw sequences (16S) after assembling and filtering were submitted to the NCBI Sequence Read Archive^1^, under accession number PRJNA844537.

#### GC-TOF/MS and identification of compounds

Rumen fluid samples were dried completely in a vacuum concentrator without heating, and then 60 μl of methoxyamination hydrochloride (20 mg/ml in pyridine) was added and incubated for 30 min at 80°C. BSTFA reagent (80 μl, 1% TMCS, v/v) was added to the sample aliquots and incubated for 1.5 h at 70°C. Samples were analyzed by an Agilent7890 gas chromatograph system coupled with a Pegasus HT time-of-flight mass spectrometer (GC-TOF/MS). The system had a DB-5MS capillary column (30 m × 250 μm inner diameters, 0.25 μm film thickness, J&W Scientific, Folsom, CA, USA) coated with 5% diphenyl cross-linked with 95% dimethylpolysiloxane. The temperature was initially kept at 50°C for 1 min, and then increased to 320°C at a rate of 10°C /min; the column was then maintained for 5 min. The temperatures of injection, transfer line, and ion source were 280°C, 270°C, and 220°C, respectively. The mass spectrometry data were acquired in full-scan mode with a mass-to-change ratio (m/z) range of 30–600 at a rate of 20 spectra/s after a solvent delay of 366 s.

Chroma TOF 4.3X software of the LECO Corporation and LECO-Fiehn Rtx5 database (Kind et al., 2009) were used for raw peak exacting, data baselines filtering, calibration of the baseline, peak alignment, deconvolution analysis, peak identification, and integration of the peak area. Both mass spectrum match and retention index match were considered in the metabolite identification.

The resulting peak number, sample name, and normalized peak area data were fed into the SIMCA software package (V14, Umetrics AB, Umea, Sweden) for principal component analysis (PCA), and orthogonal projections to latent structures-discriminant analysis (OPLS-DA). Differentially expressed metabolites (DEMs) were identified, combing Variable Importance in Projection (VIP) obtained from OPLS-DA analysis and t-test (VIP > 1 and *p* < 0.01). DEMs were further identified and validated by the Bovine Metabolome Database (BMDB^2^) and the Kyoto Encyclopedia of Genes and Genomes (KEGG^3^). The variation of different metabolites with diet concentrate ratio was identified and the horizontal, linear, and secondary effects were evaluated for the treatment groups (Zhang et al., 2020). DEM was imported into the MetaboAnalyst web server^4^ to view its metabolic pathway distribution and enrichment analysis (Xia et al., 2009).

### Correlations between microbial communities and rumen metabolites

Ruminal metabolites with VIP > 1 and *p* < 0.05, along with significantly affected microbiota were selected for use in R (v3.2.4^5^) to study changes in relevant metabolic processes. For Spearman correlation analysis, *p* values were calculated using the Psych package^6^ (author, W. Revele; publication date, 2016; version, 1.6.9), with absolute Spearman correlations of *p* > 0.05. These correlations were visualized by Pheatmap^7^ (author, R. Kolde; publication date, 2015; version 1.0.8) R and packages in Cytoscape 2.8.2 (Smoot et al., 2011).

### Statistical analysis

Statistical analyses were performed using R (v 3.6.1) and SPSS 26.0. According to Zhang et al. (2020), the linearity of the treatments was assessed by the lm function of the estimability package in R.

## Results

### Rumen fermentation parameters

Ruminal pH decreased linearly (*p* < 0.01) with increasing dietary concentrate level and had the highest value in the C50 group and the lowest value in the C80 group. Acetate concentration was significantly higher in the C80 group than in the C50 and C65 groups, and was higher in the C50 group than in the C65 group. Ruminal isobutyrate increased quadratically (*p* = 0.002), and ruminal TVFA, propionate, and butyrate concentrations increased linearly (*p* < 0.05), all of which were highest in the C80 group and lowest in the C50 group (Table 2).

**Table 2:** Effect of dietary forage to concentrate ratios on rumen fermentation parameters.

Items	Treatments^a^			SEM^b^	*p*-values		
	C50	C65	C80		Treatment	Linear	Quadratic
pH	6.49	6.37	5.94	0.077	0.001	0.001	0.411
NH_3_-N,mg/dL	12.74	12.58	13.24	0.154	0.19	0.192	0.283
MCP	2.50	2.79	2.34	0.114	0.276	0.592	0.161
TVFA, mM	76.31	80.25	90.45	2.044	0.004	0.001	0.654
VFAs, molar of TVFA							
Acetate	43.62	42.78	49.55	1.068	0.004	0.018	0.054
Propionate	15.46	17.88	19.52	0.761	0.076	0.022	0.495
Butyrate	12.49	14.13	15.31	0.479	0.038	0.01	0.455
Valerate	1.32	1.52	1.67	0.078	0.174	0.057	0.174
Isobutyrate	0.65	0.83	1.06	0.057	0.002	<0.001	0.002
Isovalerate	2.78	3.15	3.34	0.123	0.141	0.055	0.383
Acetate: Propionate	2.95	2.39	2.55	0.114	0.157	0.157	0.086

a C50, diet contained 50% of concentrate; C65, diet contained 65% of concentrate; C80, diet contained 80% of concentrate.

b SEM, standard error of the mean.

### Bacterial diversity among treatment groups

**Figure 1:**
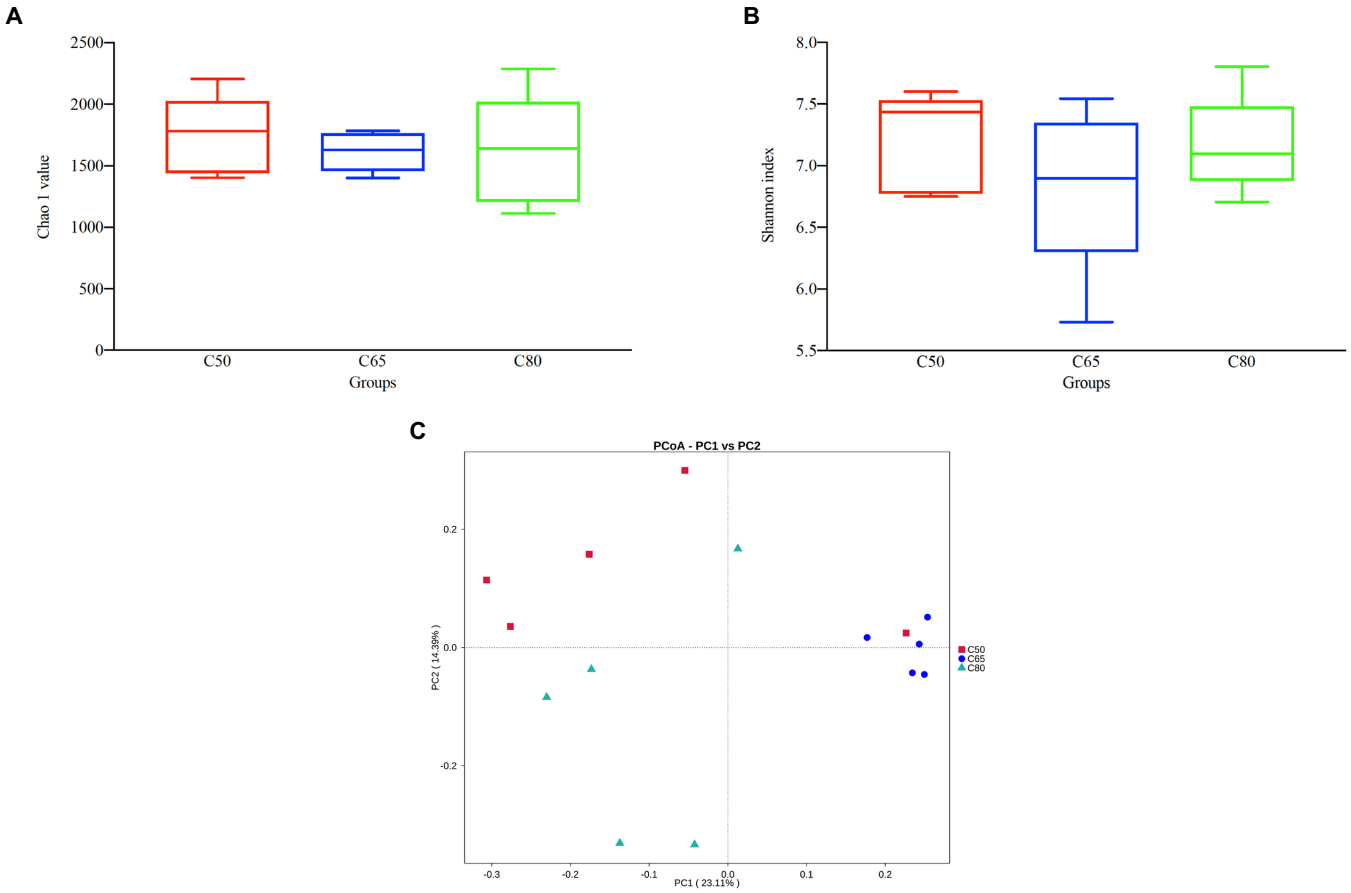
Bacterial diversity analysis of yak rumen samples for different dietary concentrate ratios. Alpha diversity between different groups’ **(A)** Chao 1 value and **(B)** Shannon index; **(C)** beta diversity principal coordinate analysis (PCoA). Differences based on concentrate to forage ratio of different diets were used.

After screening, a total of 1,157,681 high-quality 16S rRNA gene sequences were obtained from 15 rumen fluid samples. After subdividing each sample into equal sequencing depths (77,178 reads per sample) and clustering, 4,289 OTUs were obtained with 97% concordance. Microbial diversity and richness differed among the three groups according to the Shannon index and Chao1 values, indicating higher diversity and richness in the C50 group and lower diversity and richness in the C65 group, but the differences were not significant (Figures 1A,B). PCoA plots using unweighted Nifrac matrix distances, which show the diversity of the rumen microbiota for bacterial communities clustered by different concentrate to forage ratio diets, clearly showed the structure of the different bacterial communities in the three groups (*p* < 0.05). Among them, the C65 group was significantly different from the other two groups (Figure 1C), indicating that different dietary concentrate to forage ratios affected the compositions of the bacterial communities.

### Bacterial compositions among treatment groups

**Figure 2:**
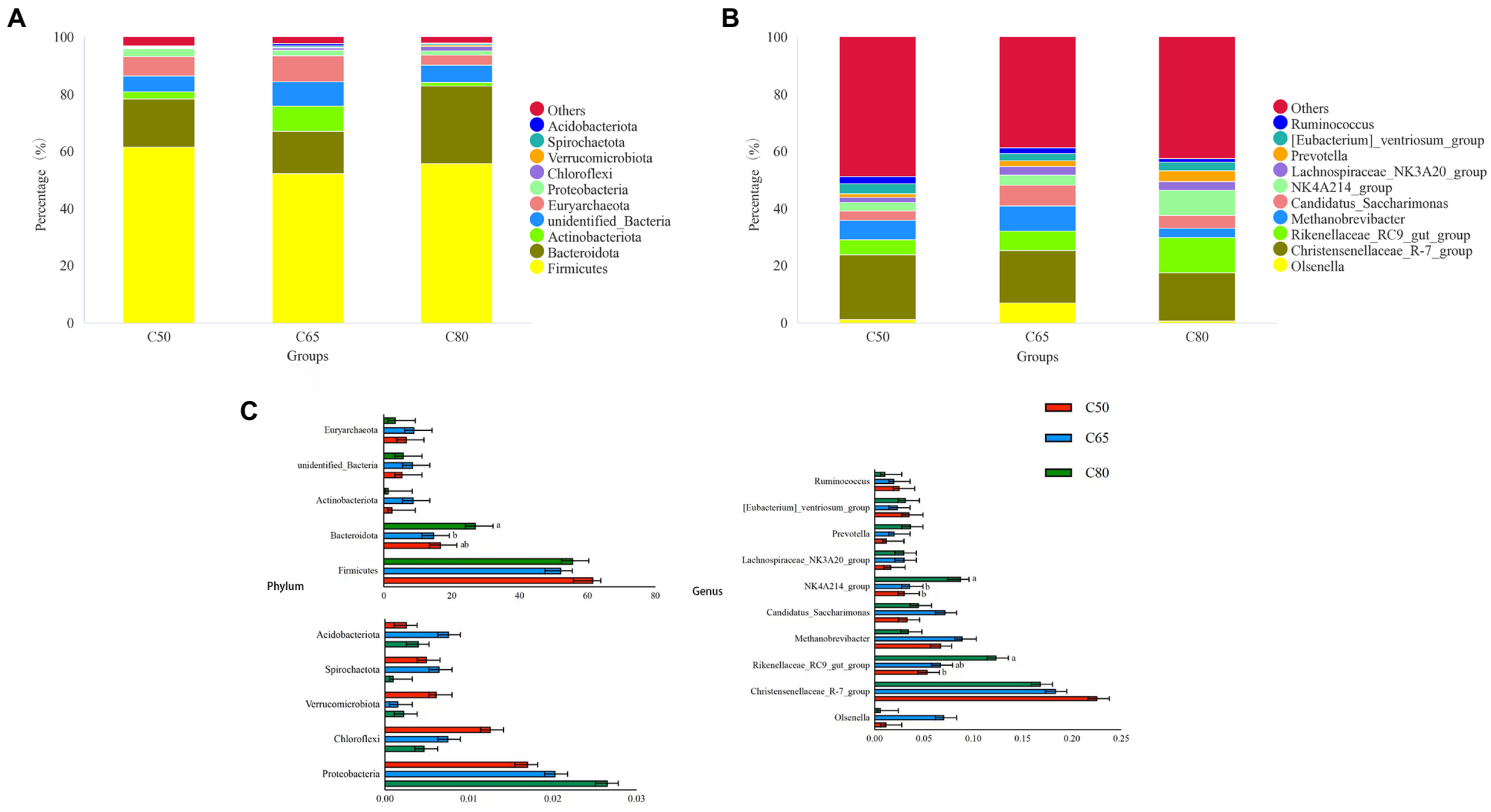
Bacterial composition of rumen samples from yaks with different dietary concentrate to forage ratio. Bacterial composition at the phylum **(A)** and genus **(B)** levels; **(C)** significantly different bacterial phylum and genus between groups, where different letters indicate significant differences between groups.

Across all groups, 59 phyla, 145 classes, 291 orders, 377 families, and 601 genera were identified. The relative abundance of dominant taxa at the phylum and genus levels is shown in Figures 2A,B. At the phylum level, the most abundant phyla were Firmicutes and Bacteroidota, which accounted for 56.56 and 19.57% of the total readings, respectively (Figure 2A). Firmicutes were enriched in the C50 group. The relative abundance of Bacteroidota in the C65 group was significantly lower than that in the C80 group (*p* < 0.05), and was followed by Actinobacteriota (4.22%), unidentified-Bacteria (6.65%), Euryarchaeota (6.43%), Proteobacteria (2.13%), Chloroflexi (0.83%), Verrucomicrobiota (0.33%), Spirochaetota (0.42%), Acidobacteriota (0.47%. At the genus level, Christensenellaceae_R-7_ group (19.27%), Rikenellaceae_RC9_gut_ group (8.15%), and Methanobrevibacter (6.36%) were the dominant genera. The content of the Christensenellace_R-7_ group was the highest. Compared with the C50 group, the Rikenellaceae_RC9_gut_ group of the C80 group was significantly richer (*p* < 0.05). The content of Methanobrevibacter in the C65 group was higher than that in the other two groups. Other genera with lower abundance included NK4A214_group (5.14%), Candidatus_Saccharimonas (5.00%), [Eubacterium]_ventriosum_group (3.02%), Olsenella (2.95%), Lachnospiraceae_NK3A20_group (2.57%), Prevotella (2.31%), and Ruminococcus (1.86%). The number of NK4A214_group in the C80 group was significantly higher than the other two groups (*p* < 0.05; Figures 2B,C).

### Identification, quantification, and statistical comparison of GC-TOF/MS metabolites in rumen

**Figure 3:**
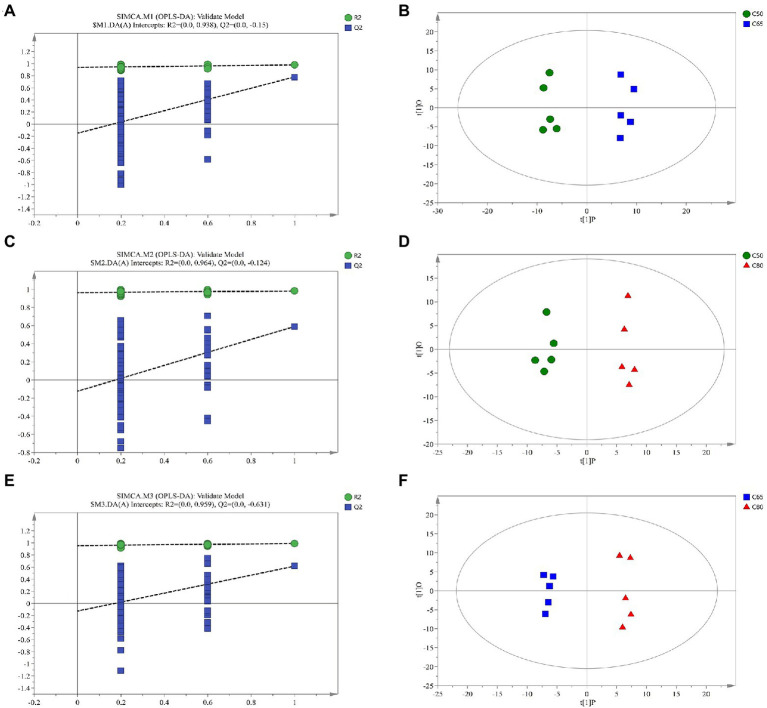
Corresponding validation plots and orthogonal projections to latent structures-discriminant analysis (OPLS-DA) score plots derived from gas chromatography time-of-flight mass spectrometer (GC-TOF/MS) metabolite profiles of yak rumen samples with different dietary concentrate ratios. Corresponding validation plots and OPLS-DA score plots for **(A,B)** group C50 vs. group C65 **(C,D)** group C50 vs. group C80, and **(E,F)** group C65 vs. group C80. C50, diets with 50% concentrate; C65, diets with 65% concentrate; C80, diets with 80% concentrate.

Supplementary Figure 1 shows the Pearson correlation coefficients between QC samples. Metabolites from rumen fluid of yaks fed with different concentrate to forage ratio diets, including QC samples, were analyzed by LC–MS, and PCA after positive and negative mode ionization showed the main unsupervised separation between the three groups (including QC samples; Supplementary Figure 2) To better distinguish the differences between the groups, OPLS-DA positive and negative mode ionization was performed to create scoring plots (Figure 3) to verify the differential metabolites between the two groups. We found that the two groups were clearly separated, with corresponding R2Y values of 0.946, 0.949, 0.96, 0.954, 0.958, and 0.978 for the OPLS DA model in C50 vs. C65, C65 vs. C80, and C50 vs. C80, respectively. All samples in the scoring plots were within 95% of the Hotelling T2 ellipse, indicating that the OPLS-DA model had satisfactory validity. The scoring plots showed clear separation and distinction between the different concentrate to forage ratio diets, indicating that the OPLS-DA model could identify differences among the groups.

### Metabolomic profiles in rumen

A total of 999 metabolites were identified. As shown in Supplementary Table 1, 107 differential metabolites were detected in the C50 vs. C65 group, 163 differential metabolites were detected in the C50 vs. C80 group, and 130 differential metabolites were detected in the C65 vs. C80 group (VIP > 1 and *p* < 0.05). Overall, 55 differential metabolites were identified among the three treatment groups, of which 20 were classified as lipids and lipid-like molecules, 14 were classified as organic acids and derivatives, 4 were classified as organoheterocyclic compounds, 5 were classified as organic oxygen compounds, 4 were classified as benzenes, 3 were classified as nucleosides, nucleotides, and analogues, 2 were classified as organic nitrogen compounds, 2 were classified as phenylpropanoids and polyketides, and 1 was classified as alkaloids and derivatives (Table 3). As shown in Table 3, changes to the dietary concentrate to forage ratio included linear and quadratic increases and decreases.

**Table 3:** Comparison of significantly changed rumen metabolites in three treatments.

Superclass	Metabolite names	Treatments^a^			SEM^b^	q-values		
		C50	C65	C80		Treatment	Linear	Quadratic
Lipids and lipid-like molecules	LPC 18:1	194691104.7	42708696.68	73549354.12	27916602.05	0.049	0.057	0.056
	(+/−)14(15)-DiHETE	7896496.452	6041559.384	10162068.62	673300.6846	0.028	0.113	0.038
	PE (18:0/18:1)	568223637.8	162,546,434	310528017.1	55846937.5	0.002	0.013	0.002
	Oleanolic acid	10860029.05	25271500.23	31586962.46	3021353.7	0.005	0.002	0.165
	Docosanoic acid	120465591.8	312561086.8	278834439.5	29895781.66	0.007	0.011	0.014
	FAHFA (18:0/20:2)	298961990.2	196,149,233	131557819.5	22268756.13	0.001	<0.001	0.192
	LPS 19:0	38712801.23	26590129.57	17910065.26	2915932.416	0.003	0.001	0.325
	1a,1b-Dihomo prostaglandin E1	6101762.044	10267200.84	15370526.54	1314266.816	0.004	0.001	0.728
	9-KODE	5199482.404	10792441.07	14612730.08	1337414.414	0.004	0.001	0.315
	FAHFA (16:0/19:2)	14935684.43	13776392.47	6942936.672	1166944.149	0.002	0.001	0.271
	8-Isoprostaglandin E2	7185522.319	7089838.662	2292831.814	802492.4605	0.006	0.004	0.175
	5-Phenylvaleric Acid	4951271.466	2543348.639	2308797.869	403568.0036	0.003	0.002	0.029
	Ursolic acid	67370520.13	262907645.3	329005364.6	43450056.48	0.023	0.009	0.209
	PC (17:2/18:5)	10851812.01	3702900.431	3488810.378	1207361.893	0.006	0.004	0.033
	(±)12(13)-DiHOME	1,108,505,784	557158819.6	1,438,674,449	148732595.2	0.035	0.289	0.024
	FAHFA (20:0/22:3)	3800547.937	4999730.565	3051165.382	356261.5261	0.067	0.339	0.044
	FAHFA (20:0/20:0)	2552261.78	3927179.497	2148783.717	298715.7078	0.024	0.499	0.011
	FAHFA (20:1/22:3)	1225762.082	3295555.767	1397935.437	296420.9791	0.001	0.688	0.000
	octadec-9-ynoic acid	142275777.6	119237853.7	358769046.8	35729092.27	0.002	0.002	0.054
	Methyl dihydrojasmonate	39830838.48	25142191.38	55470567.17	4440973.719	0.008	0.069	0.011
Organic acids and derivatives	4-Acetamidobutanoic acid	339558459.3	705539699.7	614883543.4	83776799.16	0.183	0.178	0.145
	Glycohyocholic acid Sodium salt	1121650.667	2160565.954	1293457.42	171128.2522	0.015	0.6	0.004
	Glycochenodeoxycholic acid sodium salt	138001.0444	102694.7378	210195.6813	27122.37049	0.273	0.284	0.290
	N6,N6,N6-Trimethyl-L-lysine	22992800.87	49865733.38	43396522.91	3542298.6	<0.001	0.001	0.025
	3-(1-cyano-1,2-dihydroisoquinolin-2-yl)-3-oxopropyl propionate	9809639.689	5114501.201	3930670.708	847991.9981	0.002	0.001	0.056
	2-Phenylglycine	8903501.364	12633774.31	26737460.27	2435616.441	0.001	<0.001	0.349
	FLK	4400166.146	2468044.319	1353037.499	397453.436	0.001	<0.001	0.133
	L-Cystathionine	1626295.198	1518461.996	935345.7416	109842.2172	0.009	0.004	0.395
	Val-Ser	74101368.05	56742398.55	35016005.23	5274132.354	0.002	<0.001	0.712
	Gly-Tyr-Ala	10585113.74	5431686.462	3484016.941	1071800.745	0.007	0.003	0.157
	Methylmalonic acid	6,854,758,528	10,232,838,938	6,095,994,035	644249580.7	0.008	0.517	0.003
	D-Proline	8790839.633	21238605.66	12225921.38	1605430.561	<0.001	0.122	0.000
	Methyl-2-hydroxyisobutyric acid	5627003.487	11841335.87	6323869.199	993122.1649	0.007	0.696	0.002
	5-S-cysteinyldopa	359632.8043	1151730.365	622027.8277	102360.9832	<0.001	0.082	0.000
Organoheterocyclic compounds	Uracil	2,921,751,171	2,509,346,257	2,244,999,054	152680217.3	0.196	0.079	0.617
	Roquefortine C	121793862.4	69710112.4	44521541.9	12,205,993	0.016	0.006	0.272
	Vitamin B2	9657353.128	8023761.343	5587224.07	626803.3111	0.014	0.004	0.918
	6-Dimethylaminopurine	44812097.53	20080013.45	16138724.04	4571946.438	0.008	0.004	0.072
Organic oxygen compounds	D-(+)-Mannose	1,220,583,273	2,446,884,559	1,865,896,826	184868124.8	0.012	0.08	0.006
	L-Threonic acid	75199858.64	153399194.6	83824327.61	14171561.31	0.032	0.765	0.010
	D-Galactosamine	3924881.77	9228256.303	6480804.621	697657.3705	0.001	0.029	0.000
	L-Kynurenine	7186761.226	8638010.59	9452862.755	354037.1177	0.016	0.005	0.320
	L-(−)-Glyceric acid	241945895.5	324209480.6	188479467.4	21370398.42	0.018	0.211	0.014
Benzenoids	4-Hydroxybenzoic acid	18829863.68	11260717.01	11002717.31	1431899.843	0.025	0.016	0.082
	Isohomovanillic acid	49367075.51	26081257.83	39488821.6	4000030.654	0.044	0.249	0.018
	Homovanillic acid	21776146.29	18347209.78	15121282.09	922797.1842	0.003	0.001	0.432
	2-[(carboxymethyl)(methyl)amino]-5-methoxybenzoic acid	106638622.4	324209480.6	188479467.4	21370398.42	0.085	0.321	0.060
Nucleosides, nucleotides, and analogues	2’-O-Methylguanosine	102175235.9	172895328.6	115928891.7	12413194.3	0.033	0.588	0.010
	1-Methylguanosine	13592217.39	17438884.08	5796441.008	1554608.446	0.001	0.005	0.004
	Pseudouridine	70207322.22	143837465.1	74685038.63	11250678.16	0.002	0.806	0.001
Organic nitrogen compounds	Spermidine	229394701.5	85090134.18	46356478.24	27157907.31	0.004	0.002	0.081
	2,6-di(2-thienylmethylidene)cyclohexan-1-one	92684266.52	47489798.42	47789129.5	6922044.883	0.001	0.001	0.009
Phenylpropanoids and polyketides	LSD-d3	29688333.54	19809762.59	17268311.85	1820801.817	0.003	0.001	0.068
	3-hydroxy-3,4-bis[(4-hydroxy-3-methoxyphenyl)methyl]oxolan-2-one	1912453.091	1004142.123	1065828.76	127195.7192	<0.001	<0.001	0.002
Alkaloids and derivatives	6-Acetylmorphine	12992029.47	7645667.857	5767021.69	1116835.001	0.01	0.004	0.160

a C50, diet contained 50% of concentrate; C65, diet contained 65% of concentrate; C80, diet contained 80% of concentrate.

b SEM, standard error of the mean. Significance considered at *p* < 0.05.

### Metabolic pathways of differential metabolites

**Figure 4:**
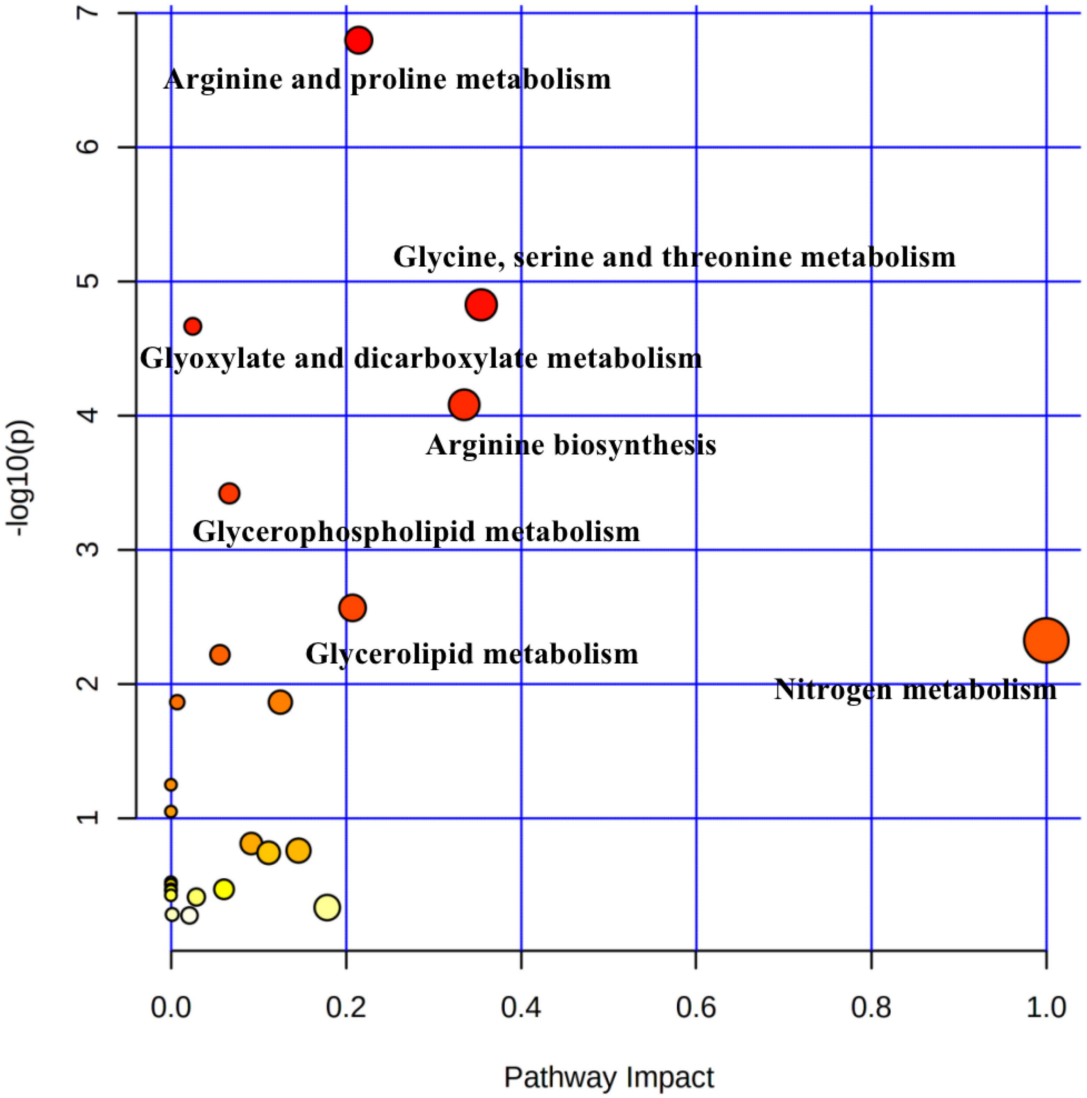
Pathway analysis of differential metabolites in rumen samples from yaks with different dietary concentrate ratios using the *Bos taurus* Kyoto Encyclopedia of Genes and Genomes (KEGG) database of MetaboAnalyst 4.0. Larger circles indicate richer pathways and darker colors indicate higher pathway impact values. The closer the color is to red, the smaller the *p*-value.

Pathway topology analysis was performed based on the identified metabolites and concentrations. Figure 4 shows seven enriched major metabolic pathways among the three treatment groups, including: arginine and proline metabolism, glycine, serine and threonine metabolism; glyoxylate and dicarboxylate metabolism; arginine biosynthesis; glycerophospholipid metabolism; glycerolipid metabolism; and nitrogen metabolism. The above pathways were identified as significantly different (*p* < 0.05). Seven metabolites (spermidine, D-proline, 4-acetamidobutanoic acid, phosphatidylcholine, 5-phenylvaleric acid, D-galactosamine, and L-threonic acid) were localized to these important pathways according to the KEGG pathway identification, and thus were identified as characteristic metabolites of 55 metabolic species.

### Correlations between the ruminal metabolomes and microbiomes

**Figure 5:**
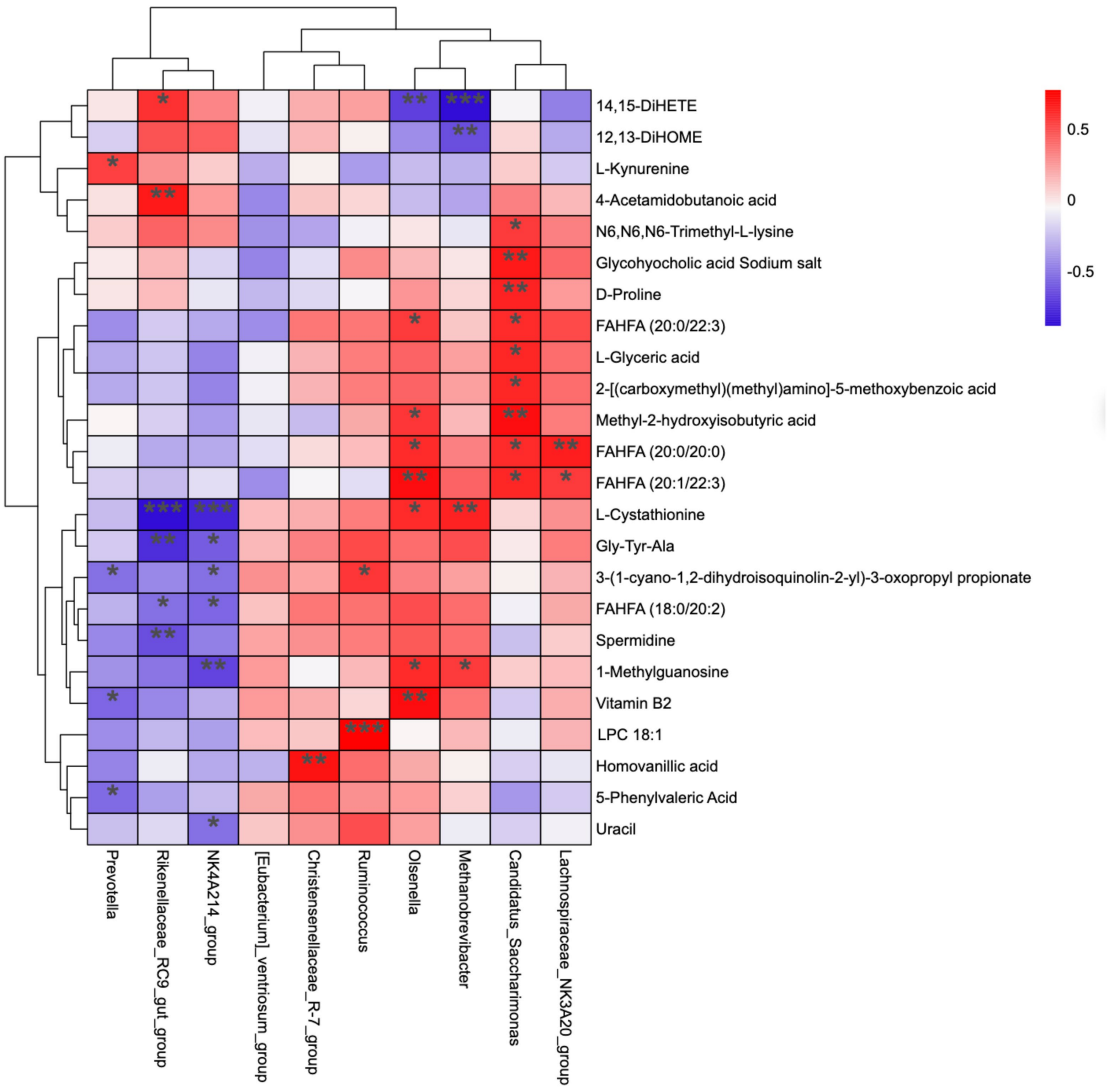
Correlation matrix between genus level and rumen differential metabolites. Each row in the figure represents a metabolite, each column represents a genus, and each lattice represents the Pearson correlation coefficient between metabolite and genus levels. Red color indicates positive correlation, while blue color indicates negative correlation. ^*^p < 0.05; ^**^p < 0.01.

Figure 5 represents the correlation analysis of rumen microbiota with metabolites. In total, 24 metabolites (from 55 differential metabolites) and 10 microbial genera were screened, and the results show that the dynamic fluctuations of certain metabolites were closely correlated with the abundance of various microbial groups. Ruminococcus was positively correlated with 3-(1-cyano-1,2-dihydroisoquinolin-2-yl)-3-oxopropyl propionate and LPC 18:1; Christensenellaceae_R-7_group was positively correlated with Homovanillic acid; NK4A214_group was positively correlated with L-cystathionine, Gly-Tyr-Ala, 3-(1-cyano-1,2-dihydroisoquinolin-2-yl)-3-oxopropyl propionate, FAHFA (18:0/20:2), 1-methylguanosine, and uracil; Rikenellaceae_RC9_gut_group was positively correlated with 14, 15-DiHET; 4-acetamido butanoic acid was positively correlated with L-cystathionine, Gly-Tyr-Ala, FAHFA (18,0/20:2), and spermidine; Prevotella was positively correlated with L-kynurenine, but negatively correlated with 3-(1-cyano-1,2-dihydroisoquinolin-2-yl)-3-oxopropyl propionate, Vitamin B2, and 5-phenylvaleric acid.

## Discussion

Dietary and nutrient compositions are important factors affecting rumen microbiota in ruminants (Yáñez-Ruiz et al., 2015). An appropriate diet concentrate to forage ratio not only improves rumen microbiota (Shen et al., 2017) but also promotes the growth and development of ruminants. In this study, we investigated the effect of dietary concentrate to forage ratio on rumen microbiota and metabolome in yaks. The results provide a reference for yak growth by regulating the relationship between the dietary concentrate to forage ratio and rumen microbiota.

A stable rumen environment is particularly important for ruminants; pH, NH_3_-N, and VFAs molar concentrations are important indicators of a stable rumen environment and reflect the status of rumen fermentation (Tomczak et al., 2019). In general, the most suitable pH for microbial growth and reproduction in the rumen is between 6.2 and 7.0, and the ecological environment of rumen microorganisms will be relatively stable within this range. When the rumen pH is below 6.0, the growth and reproduction of fibro-degrading bacteria in the rumen of ruminants is severely inhibited, affecting the digestion of fiber and inducing rumen acidosis (Grilli et al., 2016). In this study, the rumen pH of yaks in the C50 and C65 groups ranged from 6.37 to 6.49, while that of the C80 group was 5.94, indicating that rumen acidosis was induced when the percentage of concentrate increased to a certain level. Ruminal pH decreased linearly with increasing dietary concentrate level, which is consistent with the findings of Yang et al. (2000); this result may be due to the high level of dietary concentrate in the C80 group, which produced a large amount of VFAs under the degradation of rumen microorganisms, resulting in a decrease of ruminal pH.

VFAs is the end product of carbohydrates and proteins in the rumen rations of ruminants. In this study, the rumen TVFA, propionate, and butyrate concentrations increased linearly with increasing dietary concentrate level, similar to the findings of past studies (Klevenhusen et al., 2013; Giger-Reverdin et al., 2014; Polyorach et al., 2014). However, the acetic acid concentration, which was lower in the C65 group than in the C50 group and increased in the C80 group, was not consistent with the results of these past studies. This result may be due to the high level of concentrate in the C80 group, which affected the rumen environment and rumen microbial growth, resulting in abnormal rumen fermentation. Ration concentration to forage ratio increased from 50:50 to 65:35 and had a specific promotion effect on rumen TVFA content, but the ratio of concentration to forage 80:20 ratio had a negative effect instead. This study obtained the best result with a 65:35 forage to the acceptable ratio in the yaks’ diet.

The 16S rRNA gene high-throughput sequencing technology can provide a rapid and comprehensive understanding of the effect of diet concentrate to forage ratio on rumen microbiota of yaks. In this study, sequencing results revealed most of the microbiota in the samples. The Chao1 index measured the abundance of the microbiota, with a higher Chao1 index indicating greater microbiota abundance in the samples. The Shannon index measured the diversity of the microbiota, with a higher Shannon index indicating greater microbiota diversity in the samples. Rumen fluid samples from yaks fed in the C65 group had lower rumen microbiota richness and diversity compared with those fed in the C50 group, which is consistent with the results of other studies (Liu et al., 2019c; Wang et al., 2020; Ramos et al., 2021). However, rumen fluid samples from yaks reared in the C80 group showed higher abundances and diversity of rumen flora compared with yaks reared in the C65 group. It is possible that the rumen microbiota was disturbed due to the feeding of high concentrate (Krause and Oetzel, 2006; Moya et al., 2009), the rumen microbiota underwent value-added, together with the composition of the diet and the special breed of yaks, this resulted in a higher abundance and diversity of rumen microbiota in the C80 group of yaks. PCoA analysis showed that rumen microbiota clustered according to different dietary concentrate ratios and significant differences between rumen microbiota were observed, which is consistent with other studies (Hu et al., 2019; Islam et al., 2021).

In the current study, Firmicutes and Bacteroidota were the dominant phylum in all three groups of yak rumen microorganisms, similar to previous studies (Zhou et al., 2017; Bi et al., 2018). Firmicutes is mainly involved in the catabolism of fibrous material; whereas, Bacteroidota is mainly involved in the degradation of non-fibrous material (Evans et al., 2011; Reigstad and Kashyap, 2013), which is important for the nutritional metabolism of ruminants. The rumen Bacteroidota and Firmicutes were higher in the rumen of yaks in the C50 group than in the C65 group, indicating a decreasing trend of rumen Bacteroidota and Firmicutes with increasing dietary concentrate levels, consistent with the results of past studies (Nagaraja and Titgemeyer, 2007; Chiquette et al., 2008). However, the abundances of both Bacteroidota and Firmicutes in the rumen of yaks in the C80 group were higher than those in the C65 group, reflecting increasing relative abundance after rumen acidosis in yaks in the C80 group. This is consistent with the results of past studies (Mao et al., 2013; Plaizier et al., 2021), which showed relatively large increases in the numbers of bacteria associated with rumen digestion of metabolized starchy carbohydrates. This suggests that it may be necessary for rumen microbiota to maintain survival in an acidic environment by increasing the relative abundance of the phylum Bacteroidota. Combined with the experimental design, it was inferred that Bacteroidota used abundant starch substrates to reproduce rapidly over a short period of time, resulting in an increase in the relative abundance of Bacteroidota. Proteobacteria was the third most abundant phyla (Golder et al., 2014; Metzler-Zebeli et al., 2016). Proteobacteria play an important role in the formation and fermentation of biofilms and the digestion of soluble carbohydrates (Pitta et al., 2016). In this study, the relative abundance of Proteobacteria increased with increasing concentrate level, which is consistent with the findings of Wang et al. (2020). The reason for this may be due to the higher crude protein level and lower fiber content in the concentrate feeds, so it is hypothesized that the relative abundance of the Aspergillus phylum increases with increasing protein level in the feeds and is negatively correlated with the fiber level in the feeds.

At the genus level, Christensenellaceae_R-7_group, Rikenellaceae_RC9_gut_group, and Methanobrevibacter were the dominant microbiota in the rumen. The NK4A214_group, Candidatus_SaccharimonasEubacterium]_ventriosum_group, Olsenella, Lachnospiraceae_NK3A20_group, Prevotella, and Ruminococcus were also detected. The Christensenellaceae_R-7_group is described family in the phylum Firmicutes (Waters and Ley, 2019) that mainly decomposes fibrous material (Evans et al., 2011); in this study, the Christensenellaceae_R-7_group was highest in the C50 group and lowest in the C80 group. The yaks in the C80 group were fed a high level of concentrate, low level of fiber in concentrate feeds, this may account for the lowest relative abundance of the Christensenellaceae_R-7_group in this group, which is consistent with the results of Sha et al. (2021). The Rikenellaceae_RC9_gut_group belongs to the Rikenellaceae, and most are able to ferment unabsorbed polysaccharides in the host intestine, producing short-chain fatty acids (SCFAs), such as acetic acid, propionic acid, and butyric acid (Su et al., 2014). The Rikenellaceae_RC9_gut_group was significantly higher in the C80 group than in the C50 group. Because of the increase in concentrate levels, the contents of acetic acid, propionic acid and butyric acid produced by rumen fermentation in ruminants increased. Therefore that the relative abundance of Rikenellaceae_RC9_gut_group tended to increase with the increased level of concentrate. The NK4A214_group belongs to the family Rumenococcaceae, which is rich in endo-1, 4-beta-xylanas, and cellulase genes, which play an important role in the degradation of cellulose and hemicellulose; as such, their relative abundance is related to the diet concentrate to forage ratio (Koike and Kobayashi, 2009; Biddle et al., 2013). A recent study shows that NK4A214_group is positively correlated with isobutyrate and isovalerate concentrations (Liu et al., 2019b). The relative abundance of NK4A214_group was significantly higher in the C80 group than in the other two groups, similar to the results of a previously study (Chen et al., 2021), that the relative abundance of this genus increased with increasing dietary concentrate level. This can also explain the high isobutyrate and isovalerate concentrations in C80 group.

The results of principal component analysis and OPLS-DA showed significant differences in the metabolome of rumen fluid among the three groups. The study of pathway topology determined that the main metabolic pathway is nitrogen metabolism. The synthesis of rumen microbial proteins is directly related to nitrogen metabolism in the rumen (Li et al., 2019). Appropriate energy and protein levels in the rumen are more conducive to the growth and reproduction of rumen microorganisms and increase the utilization of feed protein by microorganisms (Wang et al., 2016). In the arginine and proline metabolism pathway, arginine is degraded by arginase to ornithine, which is metabolized to produce *γ*-aminobuytric acid (GABA). The 4-acetamidobutanoic acid is a derivative of GABA, which is an important mediator of arginine and proline metabolism (Gao et al., 2017); GABA acts as an excitatory transmitter in the early stages of life and is involved in brain development (Hasegawa et al., 2018). In our study, 4-acetamidobutanoic acid was positively correlated with Rikenellaceae_RC9_gut_group, which belongs to Rikenellaceae and produces short-chain fatty acids (SCFAs) to provide more energy, which has a positive effect on arginine and proline metabolism. Proline is a cyclic amino acid that enters the peptide chain and undergoes hydroxylation to form 4-hydroxyproline, an important component of animal proteins. In ruminants, arginine and proline are among the amino acids rich in casein, which promotes the digestion of cellulose by rumen microorganisms and play an important role in protein synthesis, metabolism, and nutrition (Amos et al., 1971; Bruckental and Alumot, 1984; Wu et al., 2011). In this study, the highest contents of 4-acetamidobutanoic acid and proline were found in the C65 group. One possible explanation is that the C65 group produced higher concentrations of proline which is more favorable for rumen amino acid metabolism and organism protein synthesis. In addition, Candidatus_Saccharimonas is a potential beneficial bacterium, which is closely related to fiber degradation and can produce cellulase in the rumen to degrade fiber-like substances such as cellobiose, and is a typical class of fiber-degrading bacteria (Liu et al., 2019a), whose increased content can improve fiber digestibility (Patra and Yu, 2015), which can promote the digestion and utilization of rumen nutrients to a certain extent. Correlation analysis showed that proline was positively correlated with Candidatus_Saccharimonas; therefore, the C65 group increased the concentration of proline in the rumen by increasing the relative abundance of Candidatus_Saccharimonas.

Glyoxylate and dicarboxylate metabolism, along with glycine, serine, and threonine metabolism are involved in the regulation of energy metabolism and immune regulation in the body; both metabolic pathways are associated with organic acids and provide important energy metabolic precursors for entry into the citric acid cycle (Yu et al., 2017). The metabolites L-threonic acid and PC (17,2/18:5) are present in this metabolic pathway, and threonine is converted to glycin and serine, which are key metabolites for protein synthesis (Laura et al., 2019). Phosphatidylcholine (PC) is an important component of the cellular lipid bilayer and belongs to a glycerophospholipid. Choline is a major component of PC, and sufficient PC facilitates lipid transport (Shiau and Cho, 2002; Yeh et al., 2013). PC acts as a choline “storage “molecule, and choline can be metabolized to organic osmolytes *via* the glycine, serine, and threonine metabolism pathway (Athamena et al., 2011; Jiang et al., 2019).

Moreover, this metabolite is also involved in the glycerophospholipid metabolism pathway (Mohamad et al., 2015). Lipid metabolism includes glycerophospholipid metabolism and glycerolipid metabolism. Lipid and lipid-like molecules in gut microbiota are associated with lipid and lipid-like metabolites that may affect lipid metabolism by increasing substrates for energy metabolism in the liver and surrounding tissues (Bäckhed and Crawford, 2010). In the present study, we found that the concentration of L-threonic acid tended to increase and then decrease, with the highest concentration in the C65 group; the concentration of PC (17:2/18:5) increased linearly with increasing concentrate levels. Our results suggest that metabolites related to lipid metabolism play an important role in the growth and rumen health of yaks. Increasing concentrate level increases the concentration of threonine in the rumen of yaks and promotes glycine, serine, and threonine metabolism; in turn, this improves the growth performance of yaks.

Arginine is a conditionally essential amino acid that plays an important role in the physiological function and metabolic regulation of animal organisms. Arginine promotes urea synthesis, regulates ammonia metabolism through the urea cycle, and improves reproductive performance, lactation function, immunity, growth performance, meat quality, and feed utilization (Chacher et al., 2013; Yohan et al., 2016; Zy et al., 2020). Spermidine is a derivative of arginine produced through the ornithine metabolism pathway and is a polyamine with important physiological functions (Minois et al., 2013). In the present study, the concentration of spermidine showed a linear increase with increasing concentration level, and was negatively correlated with Rikenellaceae_RC9_gut_group. This indicates that the concentration of arginine is affected by an increasing level of concentrate; therefore, raising the level of concentrate can promote arginine biosynthesis and contribute to the growth performance of yaks.

The results of this study show that there is a complex relationship between yak rumen microbes and their metabolites, both of which are influenced by diet concentrate to forage ratios. The results showed that different dietary concentrate ratios had major effects on rumen fermentation, microbial composition and metabolic functions in yaks. According to the results of this experiment, we found that the increase of concentrate level had a certain promotion effect on TVFA content in yak rumen and reduced the relative abundance of bacteria related to fiber degradation, increased the concentration of threonine in yak rumen and promoted the metabolism of glycine, serine and threonine, and the growth of yak was improved, the high ratio of forage to concentrate in the diet had negative effects. In summary, this study combined microbiomic and metabolomic analysis of the correlations among microbiota and differential metabolites in the rumen of yaks under different dietary concentrate ratios. The results offer new insight into yak rumen microbiota and metabolites, and provide guidance for optimal yak diet concentrate ratios to improve the growth performance of housed yaks.

## Data availability statement

The datasets presented in this study can be found in online repositories. The names of the repository/repositories and accession number(s) can be found in the article/Supplementary material.

## Ethics statement

All procedures in this study were approved by the Institutional Animal Care and Use Committee of Qinghai University.

## Author contributions

SW, SC, and KP contributed to conception and design of the study. KP, SW, and XW collected the samples. SW, KP, YY, and SC conducted relevant experiments. KP and SW organized the database. XW and SL performed the statistical analysis. KP wrote the first draft of the manuscript. XW, YY, SL, and SC wrote sections of the manuscript. All authors contributed to the article and approved the submitted version.

## Funding

This research was funded by the Qinghai Provincial Science and Technology Department of China (grant no. 2020-ZJ-935Q) and Qinghai Province Thousands of High-end Innovative Talents Plan.

## Conflict of interest

The authors declare that the research was conducted in the absence of any commercial or financial relationships that could be construed as a potential conflict of interest.

## Publisher’s note

All claims expressed in this article are solely those of the authors and do not necessarily represent those of their affiliated organizations, or those of the publisher, the editors and the reviewers. Any product that may be evaluated in this article, or claim that may be made by its manufacturer, is not guaranteed or endorsed by the publisher.

## Footnotes

^1^ http://drive5.com/uparse/

^1^ https://www.ncbi.nlm.nih.gov/bioproject/PRJNA844537

^2^ https://bovinedb.ca

^3^ https://www.genome.jp/kegg/

^4^ https://www.metaboanalyst.ca

^5^ http://www.r-project.org/

^6^ https://cran.r-project.org/web/packages/psych/

^7^ https://cran.r-project.org/web/packages/pheatmap/index.html

## References

[ref1] Ahmad A. A. Yang C. Zhang J. Kalwar Q. Ding X. (2020). Effects of dietary energy levels on rumen fermentation, microbial diversity, and feed efficiency of yaks (*Bos grunniens*). Front. Microbiol. 11:625. doi: 10.3389/fmicb.2020.00625 32670204 PMC7326093

[ref2] Amos H. E. Little C. O. Mitchell G. E. Jr. (1971). Proline utilization during cellulose fermentation by rumen microorganisms. Agric. Food Chem. 19:51. doi: 10.1021/jf60173a051 5540744

[ref3] AOAC (2007). Official Methods of Analysis. Gaithersburg, MD: Association of Official Analytical Chemists.

[ref4] Athamena A. Brichon G. Trajkovic-Bodennec S. Péqueux A. Chapelle S. Bodennec J.. (2011). Salinity regulates N-methylation of phosphatidylethanolamine in euryhaline crustaceans hepatopancreas and exchange of newly-formed phosphatidylcholine with hemolymph. Compr. Physiol. 181, 731–740. doi: 10.1007/s00360-011-0562-6 21416254

[ref5] Bäckhed F. Crawford P. A. (2010). Coordinated regulation of the metabolome and lipidome at the host-microbial interface. Biochim. Biophys. Acta 1801, 240–245. doi: 10.1016/j.bbalip.2009.09.009 19782151 PMC2823845

[ref6] Bi Y. L. Zeng S. Q. Zhang R. Diao Q. Y. Tu Y. (2018). Effects of dietary energy levels on rumen bacterial community composition in Holstein heifers under the same forage to concentrate ratio condition. BMC Microbiol. 18:69. doi: 10.1186/s12866-018-1213-9 29996759 PMC6042446

[ref7] Biddle A. Stewart L. Blanchard J. Leschine S. (2013). Untangling the genetic basis of fibrinolytic specialization by lachnospiraceae and ruminococcaceae in diverse gut communities. Diversity 5, 389627–389640. doi: 10.3390/d5030627

[ref8] Broderick G. A. Kang J. H. (1980). Automated simultaneous determination of ammonia and total amino acids in ruminal fluid and in vitro medial. Dairy Sci. 63, 64–75. doi: 10.3168/jds.S0022-0302(80)82888-8 7372898

[ref9] Bruckental I. Alumot E. (1984). Proline supply for efficient nitrogen-utilization by lactating ruminants. Can. J. Anim. Sci. 64, 285–286. doi: 10.4141/cjas84-263

[ref10] Cao Z. J. Li S. L. Xing J. J. Ma M. Wang L. L. (2008). Effects of maize grain and lucerne particle size on ruminal fermentation, digestibility and performance of cows in midlactation. Anim. Physiol. Anim. Nutr. 92, 157–167. doi: 10.1111/j.1439-0396.2007.00721.x 18336412

[ref11] Caporaso J. G. Kuczynski J. Stombaugh J. Bittinger K. Bushman F. D. Costello E. K.. (2010). QIIME allows analysis of high-throughput community sequencing data. Nat. Methods 7, 335–336. doi: 10.1038/nmeth.f.303 20383131 PMC3156573

[ref12] Chacher B. Liu H. Y. Wang D. M. Liu J. X. (2013). Potential role of N-carbamoyl glutamate in biosynthesis of arginine and its significance in production of ruminant animals. J. Anim. Sci. Biotechn. 4:16. doi: 10.1186/2049-1891-4-16 PMC3627613 23575433

[ref13] Chen H. Wang C. Huasai S. Chen A. (2021). Effects of dietary forage to concentrate ratio on nutrient digestibility, ruminal fermentation and rumen bacterial composition in Angus cows. Sci. Rep. 11, 17023. doi: 10.1038/s41598-021-96580-5 34426627 PMC8382751

[ref14] Chiquette J. Allison M. J. Rasmussen M. A. (2008). *Prevotella bryantii* 25a used as a probiotic in early-lactation dairy cows: effect on ruminal fermentation characteristics, milk production, and milk composition. J. Dairy Sci. 91, 3536–3543. doi: 10.3168/jds.2007-0849 18765612

[ref15] Edgar R. C. (2010). Search and clustering orders of magnitude faster than BLAST. Bioinformatics 26, 2460–2461. doi: 10.1093/bioinformatics/btq461 20709691

[ref16] Evans N. J. Brown J. M. Murray R. D. Getty B. Birtles R. J. Hart C. A.. (2011). Characterization of novel bovine gastrointestinal tract treponema isolates and comparison with bovine digital dermatitis treponemes. Appl. Environ. Microbiol. 77, 138–147. doi: 10.1128/AEM.00993-10 21057019 PMC3019712

[ref17] Fan Q. S. Wanapat M. Hou F. J. (2021). Chemical composition of milk and rumen microbiome diversity of yak, impacting by herbage grown at different phenological periods on the Qinghai-Tibet plateau. Animals 2021, 1030. doi: 10.3390/ani10061030 PMC7341253 32545764

[ref18] Gao X. X. Liang M. L. Fang Y. Zhao F. Tian J. S. Zhang X.. (2017). Deciphering the differential effective and toxic responses of Bupleuri Radix following the induction of chronic unpredictable mild stress and in healthy rats based on serum metabolic profiles. Front. Pharmacol. 8:995. doi: 10.3389/fphar.2017.00995 29379441 PMC5775221

[ref19] Giger-Reverdin S. Rigalma K. Desnoyers M. Sauvant D. Duvaux-Ponter C. (2014). Effect of concentrate level on feeding behavior and rumen and blood parameters in dairy goats: relationships between behavioral and physiological parameters and effect of between-animal variability. J. Dairy Sci. 97, 4367–4378. doi: 10.3168/jds.2013-7383 24952476

[ref20] Golder H. M. Denman S. E. McSweeney C. Wales W. J. Auldist M. J. Wright M. M.. (2014). Effects of partial mixed rations and supplement amounts on milk production and composition, ruminal fermentation, bacterial communities, and ruminal acidosis. J. Dairy Sci. 97, 5763–5785. doi: 10.3168/jds.2014-8049 24997657

[ref22] Grilli D. J. Fliegerová K. Kopečný J. Lama S. P. Egea V. Sohaefer N.. (2016). Analysis of the rumen bacterial diversity of goats during shift from forage to concentrate diet. Anaerobe 42, 17–26. doi: 10.1016/j.anaerobe.2016.07.002 27417742

[ref23] Hasegawa Y. Curtis B. Yutuc V. Rulien M. Morrisroe K. Watkins K.. (2018). Microbial structure and function in infant and juvenile rhesus macaques are primarily affected by age, not vaccination status. Sci. Rep. 8:15867. doi: 10.1038/s41598-018-34019-0 30367140 PMC6203732

[ref24] Heinken A. Sahoo S. Fleming R. M. T. Thiele I. (2014). Systems-level characterization of a host-microbe metabolic symbiosis in the mammalian gut. Gut Microbes 4, 28–40. doi: 10.4161/gmic.22370 PMC3555882 23022739

[ref25] Hu R. Zou H. Wang Z. Cao B. Peng Q. Jing X.. (2019). Nutritional interventions improved rumen functions and promoted compensatory growth of growth-retarded yaks as revealed by integrated transcripts and microbiome analyses. Front. Microbiol. 10, 318. doi: 10.3389/fmicb.2019.00318 30846981 PMC6393393

[ref26] Islam M. Kim S. Ramos S. C. Mamuad L. L. Son A.-R. Zhong T. Y. (2021). Holstein and Jersey steers differ in rumen microbiota and enteric methane emissions even fed the same total mixed ration. Front. Microbiol. 12:601061. doi: 10.3389/fmicb.2021.601061 33868186 PMC8044996

[ref27] Jiang W. W. Tian X. L. Fang Z. H. Li L. Dong S. L. Li H. D.. (2019). Metabolicresponses in the gills of tongue sole (*Cynoglossus semilaevis*) exposed to salinity stress using NMR-based metabolomics. Sci. Total Environ. 653, 465–474. doi: 10.1016/j.scitotenv.2018.10.404 30412891

[ref28] Kind T. Wohlgemuth G. Lee D. Y. Lu Y. Palazoglu M. Shahbaz S.. (2009). FiehnLib: mass spectral and retention index libraries for metabolomics based on quadrupole and time-of-flight gas chromatography/mass spectrometry. Anal. Chem. 81, 10038–10048. doi: 10.1021/ac9019522 19928838 PMC2805091

[ref29] Klevenhusen F. Hollmann M. Podstatzky-Lichtenstein L. Krametter-Frotscher R. Aschenbach J. R. Zebeli Q. (2013). Feeding barley grain-rich diets altered electrophysiological properties and permeability of the ruminal wall in a goat model. J. Dairy Sci. 96, 2293–2302. doi: 10.3168/jds.2012-6187 23403198

[ref30] Koike S. Kobayashi Y. (2009). Fibrolytic rumen bacteria: their ecology and functions. Asian Aust. J. Anim. Sci. 40, 1141–1147. doi: 10.5713/ajas.2009.r.01

[ref31] Krause K. M. Oetzel G. R. (2006). Understanding and preventing subacute ruminal acidosis in dairy herds: a review. Anim. Feed Sci. Technol. 126, 215–236. doi: 10.1016/j.anifeedsci.2005.08.004

[ref21] Laura G. Amanda G. Marcio G. Uiseli O. Touchette K. J. (2019). Evaluation of the optimal standardized ileal digestible threonine: lysine ratio in lactating sow diets. J. Anim. Sci. 97, 2972–2978. doi: 10.1093/jas/skz181 31125085 PMC6606533

[ref32] Li W. Gelsinger S. Edwards A. Riehle C. Koch D. (2019). Transcriptome analysis of rumen epithelium and meta-transcriptome analysis of rumen epimural microbial community in young calves with feed induced acidosis. Sci. Rep. doi: 10.1038/s41598-019-40375-2 PMC6426933 30894588

[ref33] Liu Y. Z. Lang M. Zhen Y. G. Chen X. Sun Z. Zhao W.. (2019a). Effects of yeast culture supplementation and the ratio of non-structural carbohydrate to fat on growth performance, carcass traits and the fatty acid profile of the longissimus dorsi muscle in lambs. J. Anim. Physiol. Anim. Nutr. 103, 1274–1282. doi: 10.1111/jpn.13128 31149756

[ref34] Liu C. Wu H. Liu S. J. Chai S. T. Meng Q. X. Zhou Z. M. (2019b). Dynamic alterations in yak rumen Bacteria community and metabolome characteristics in response to feed type. Front. Microbiol. 10, 1116. doi: 10.3389/fmicb.2019.01116 31191470 PMC6538947

[ref35] Liu H. J. Xu T. W. Xu S. X. Ma L. Zhao X. Q. (2019c). Effect of dietary concentrate to forage ratio on growth performance, rumen fermentation and bacterial diversity of Tibetan sheep under barn feeding on Qinghai-Tibetan plateau. PeerJ. 7:e7462. doi: 10.7287/peerj.preprints.27807v2 31404417 PMC6686838

[ref36] Long R. J. Ding L. M. Shang Z. H. Guo X. H. (2008). The yak grazing system on the Qinghai-Tibetan plateau and its status. The. Rangel. J. 30, 241–246. doi: 10.1071/RJ08012

[ref37] Mao S. Y. Zhang M. L. Liu J. H. Zhu W. Y. (2015). Characterising the bacterial microbiota across the gastrointestinal tracts of dairy cattle: membership and potential function. Sci. Rep. 5:16116. doi: 10.1038/srep16116 26527325 PMC4630781

[ref38] Mao S. Y. Zhang R. Y. Wang D. S. Zhu W. Y. (2013). Impact of subacute ruminal acidosis (SARA) adaptation on rumen microbiota in dairy cattle using pyrosequencing. Anaerobe 24, 12–19. doi: 10.1016/j.anaerobe.2013.08.003 23994204

[ref39] Metzler-Zebeli B. U. Khol-Parisini A. Gruber L. Zebeli Q. (2016). Microbial populations and fermentation profiles in rumen liquid and solids of Holstein cows respond differently to dietary barley processing. J. Appl. Microbiol. 119, 1502–1514. doi: 10.1111/jam.12958 26399366

[ref40] Minois N. Carmona-Gutierrez D. Madeo F. (2013). Polyamines in aging and disease. Aging 8:3, 716–732. doi: 10.18632/aging.100361 PMC3184975 21869457

[ref41] Mohamad N. Ismet R. I. Rofiee M. Bannur Z. Hennessy T. Selvaraj M. (2015). Metabolomics and partial least square discriminant analysis to predict history of myocardial infarction of self-claimed healthy subjects: validity and feasibility for clinical practice. J. Clin. Bioinforma. 5:3. doi: 10.1186/s13336-015-0018-4 25806102 PMC4371619

[ref42] Moya D. Calsamiglia S. Ferret A. Blanch M. Fandiño J. I. Castillejos L.. (2009). Effects of dietary changes and yeast culture (*Saccharomyces cerevisiae*) on rumen microbial fermentation of Holstein heifers. J. Anim. Sci. 87, 2874–2881. doi: 10.2527/jas.2008-1446 19542509

[ref43] Nagaraja T. G. Titgemeyer E. C. (2007). Ruminal acidosis in beef cattle: the current microbiological and nutritional outlook. J. Dairy Sci. 90, E17–E38. doi: 10.3168/jds.2006-478 17517750

[ref44] Patra A. K. Yu Z. (2015). Essential oils affect populations of some rumen bacteria in vitro as revealed by microarray (RumenBactArray) analysis. Front. Microbiol. 6:297. doi: 10.3389/fmicb.2015.00297 25914694 PMC4392297

[ref45] Pitta D. W. Pinchak W. E. Indugu N. Vecchiarelli B. Sinha R. Fulford J. D. (2016). Metagenomic analysis of the rumen microbiome of steers with wheat-induced frothy bloat. Front. Microbiol. 7:689. doi: 10.3389/fmicb.2016.00689 27242715 PMC4863135

[ref46] Plaizier J. C. Danscher A. M. Azevedo P. A. Derakhshani H. Khafipour E. (2021). A grain-based SARA challenge affects the composition of epimural and mucosa-associated bacterial communities throughout the digestive tract of dairy cows. Animals 11:1658. doi: 10.3390/ani11061658 34199660 PMC8227306

[ref47] Polyorach S. Wanapat M. Cherdthong A. (2014). Influence of yeast fermented cassava chip protein (YEFECAP) and roughage to concentrate ratio on ruminal fermentation and microorganisms using gas production technique. Asian-Australas J. Anim. Sci. 27, 36–45. doi: 10.5713/ajas.2013.13298 25049924 PMC4093292

[ref48] Pruesse E. Quast C. Knittel K. Fuchs B. M. Ludwig W. Peplies J.. (2007). SILVA: a comprehensive online resource for quality checked and aligned ribosomal RNA sequence data compatible with ARB. Nucleic Acids Res. 21, 7188–7196. doi: 10.1093/nar/gkm864 PMC2175337 17947321

[ref49] Qiu Q. H. Gao C. Y. Aziz Ur Rahman M. Cao B. H. Su H. W. (2020). Digestive ability, physiological characteristics, and rumen bacterial community of Holstein finishing steers in response to three nutrient density diets as fattening phases advanced. Microorganisms 8:335. doi: 10.3390/microorganisms8030335 PMC7142484 32120877

[ref50] Ramos S. C. Chang D. J. Mamuad L. L. Ho S. Sang S. L. (2021). Diet transition from high-forage to high-concentrate alters rumen bacterial community composition, epithelial transcriptomes and ruminal fermentation parameters in dairy cows. Animals 11:838. doi: 10.3390/ani11030838 33809588 PMC8002347

[ref51] Reigstad C. S. Kashyap P. C. (2013). Beyond phylotyping: understanding the impact of gut microbiota on host biology. Neurogastroenterol. Motil. 25, 358–372. doi: 10.1111/nmo.12134 23594242 PMC4524550

[ref52] Saleem F. Bouatra S. Guo A. C. Psychogios N. Mandal R. M. S. Dunn S. M.. (2013). The bovine ruminal fluid metabolome. Metabolomics 9, 360–378. doi: 10.1007/s11306-012-0458-9

[ref53] Schloss P. D. Westcott S. L. Ryabin T. Hall J. R. Hartmann M. Hollister E.. (2009). Introducing mothur: open-source, platform-independent, community-supported software for describing and comparing microbial communities. Appl. Environ. Microbiol. 75:7537. doi: 10.1128/AEM.01541-09 19801464 PMC2786419

[ref54] Sha Y. Z. Hu J. Shi B. G. Dingkao R. Q. Liu X. (2021). Supplementary feeding of cattle-yak in the cold season alters rumen microbes, volatile fatty acids, and expression of sglt1 in the rumen epithelium. PeerJ. 9:11048. doi: 10.7717/peerj.11048 PMC7982075 33777531

[ref55] Shen H. Lu Z. Y. Xu Z. H. Shen Z. M. (2017). Diet-induced reconstruction of mucosal microbiota associated with alterations of epithelium lectin expression and regulation in the maintenance of rumen homeostasis. Sci. Rep. 7:3941. doi: 10.1038/s41598-017-03478-2 28638072 PMC5479827

[ref56] Shiau S. Y. Cho W. H. (2002). Choline requirements of grass shrimp (*Penaeus monodon*) as affected by dietary lipid level. Anim. Sci. 75, 97–102. doi: 10.1017/S1357729800052875

[ref57] Smoot M. E. Ono K. Ruscheinski J. Wang P. L. Ideker T. (2011). Cytoscape 2.8: new features for data integration and network visualization. Bioinformatics 27, 431–432. doi: 10.1093/bioinformatics/btq675 21149340 PMC3031041

[ref58] Su X. L. Tian Q. Zhang J. Yuan X. Z. Shi X. S. Guo R. B.. (2014). Acetobacteroides hydrogenigenes gen. Nov., sp. nov., an anaerobic hydrogen-producing bacterium in the family Rikenellaceae isolated from a reed swamp. Int. J. Sys. Evol. 64, 2986–2991. doi: 10.1099/ijs.0.063917-0 24899658

[ref59] Tomczak D. J. Samuelson K. L. Jennings J. S. Richeson J. T. (2019). Oral hydration therapy with water and bovine respiratory disease incidence affects rumination behavior, rumen pH, and rumen temperature in high-risk, newly received beef calves. J. Anim. Sci. 5, 2015–2024. doi: 10.1093/jas/skz102 PMC6488335 30911760

[ref60] Vansoest P. J. Robertson J. B. Lewis B. A. (1991). Methods for dietary fiber, neutral detergent fiber, and nonstarch polysaccharides in relation to animal nutrition. J. Dairy Sci. 74, 3583–3597. doi: 10.3168/jds.S0022-0302(91)78551-2 1660498

[ref61] Wang X. J. Ao C. J. Khas-Erdene L. S. W. Chen B. Zhang F. Q. Zhang Y.. (2016). Effects of infusing milk precursors into the artery on rumen fermentation in lactating cows. Anim. Nutr. 6:2. doi: 10.1016/j.aninu.2016.03.002 PMC5941015 29767029

[ref62] Wang L. Li Y. Zhang Y. Wang L. (2020). The effects of different concentrate-to-forage ratio diets on rumen bacterial microbiota and the structures of Holstein cows during the feeding cycle. Animals 10:957. doi: 10.3390/ani10060957 PMC7341334 32486436

[ref63] Waters J. L. Ley R. E. (2019). The human gut bacteria Christensenellaceae are widespread, heritable, and associated with health. BMC Biol. 17:4. doi: 10.1186/s12915-019-0699-4 31660948 PMC6819567

[ref64] Wu G. Bazer F. W. Burghardt R. C. Johnson G. A. Kim S. W. Knabe D. A.. (2011). Proline and hydroxyproline metabolism: implications for animal and human nutrition. Amino Acids 40, 1053–1063. doi: 10.1007/s00726-010-0715-z 20697752 PMC3773366

[ref65] Wu D. W. Vinitchaikul P. Deng M. Y. Zhang G. G. Sun L. Y. Wang H. X.. (2021). Exploration of the effects of altitude change on bacteria and fungi in the rumen of yak (*Bos grunniens*). Arch. Microbiol. 2021.203, 835–846. doi: 10.1007/s00203-020-02072-x 33070234

[ref66] Xia J. Psychogios N. Young N. Wishart D. S. (2009). MetaboAnalyst: a web server for metabolomic data analysis and interpretation. Nucleic Acids Res. 37, W652–W660. doi: 10.1093/nar/gkp356 19429898 PMC2703878

[ref67] Yáñez-Ruiz D. R. Abecia L. Newbold C. J. (2015). Manipulating rumen microbiome and fermentation through interventions during early life: a review. Front. Microbiol. 6:1133. doi: 10.3389/fmicb.2015.01133 26528276 PMC4604304

[ref68] Yang W. Z. Beauchemin K. A. Rode L. M. (2000). Effects of barley grain processing on extent of digestion and milk production of lactating dairy cows. J. Dairy Sci. 83, 554–568. doi: 10.3168/jds.S0022-0302(00)74915-0 10750114

[ref69] Ye H. Liu J. H. Feng P. F. Zhu W. Y. Mao S. Y. (2016). Grain-rich diets altered the colonic fermentation and mucosa-associated bacterial communities and induced mucosal injuries in goats. Sci. Rep. 6:20329. doi: 10.1038/srep20329 26841945 PMC4740883

[ref70] Yeh S. P. Shiu P. J. Guei W. C. Lin Y. H. Liu C. H. (2013). Improvement in lipid metabolism and stress tolerance of juvenile giant grouper, *Epinephelus lanceolatus* (Bloch), fed supplemental choline. Aquac. Res. 46, 1810–1821. doi: 10.1111/are.12334

[ref71] Yohan P. Ju-Hwan P. Suryeon P. Song L. Kwan C. Dae-Duk K.. (2016). Enhanced cellular uptake and pharmacokinetic characteristics of doxorubicin-valine amide prodrug. Molecules 21:1272. doi: 10.3390/molecules21101272 PMC6274118 27669201

[ref72] Yu M. Jia H. M. Zhou C. Yang Y. Sun L. L. Zou Z. M. (2017). Urinary and fecal metabonomics study of the protective effect of Chaihu-Shu-Gan-San on antibiotic-induced gut microbiota dysbiosis in rats. Sci. Rep. 7:46551. doi: 10.1038/srep46551 28425490 PMC5397834

[ref73] Zhang J. Shi H. Wang Y. J. Li S. L. Cao Z. J. Ji S. K.. (2017). Effect of dietary forage to concentrate ratios on dynamic profile changes and interactions of ruminal microbiota and metabolites in Holstein heifers. Front. Microbiol. 8:2206. doi: 10.3389/fmicb.2017.02206 29170660 PMC5684179

[ref74] Zhang J. Shi H. T. Wang Y. C. Li S. L. Cao Z. J. Yang H. J.. (2020). Carbohydrate and amino acid metabolism and oxidative status in Holstein heifers precision-fed diets with different forage to concentrate ratios. Animal 14, 2315–2325. doi: 10.1017/S1751731120001287 32602427

[ref75] Zhou Z. M. Fang L. Meng Q. X. Li S. L. Chai S. T. Liu S. J.. (2017). Assessment of ruminal bacterial and archaeal community structure in yak (*Bos grunniens*). Front. Microbiol. 8:179. doi: 10.3389/fmicb.2017.00179 28223980 PMC5293774

[ref76] Zy A. Jkh B. Sfl A. (2020). Methionine nutrition in swine and related monogastric animals: beyond protein biosynthesis. Anim. Feed Sci. Technol. 8:114608. doi: 10.1016/j.anifeedsci.2020.114608

