# Supplementary figures and images for: Dietary forage to concentrate ratios impact on yak ruminal microbiota and metabolites
Source: Front Microbiol. 2022 Aug 11;13:964564. doi: 10.3389/fmicb.2022.964564 (PMC9410728; doi:10.3389/fmicb.2022.964564)

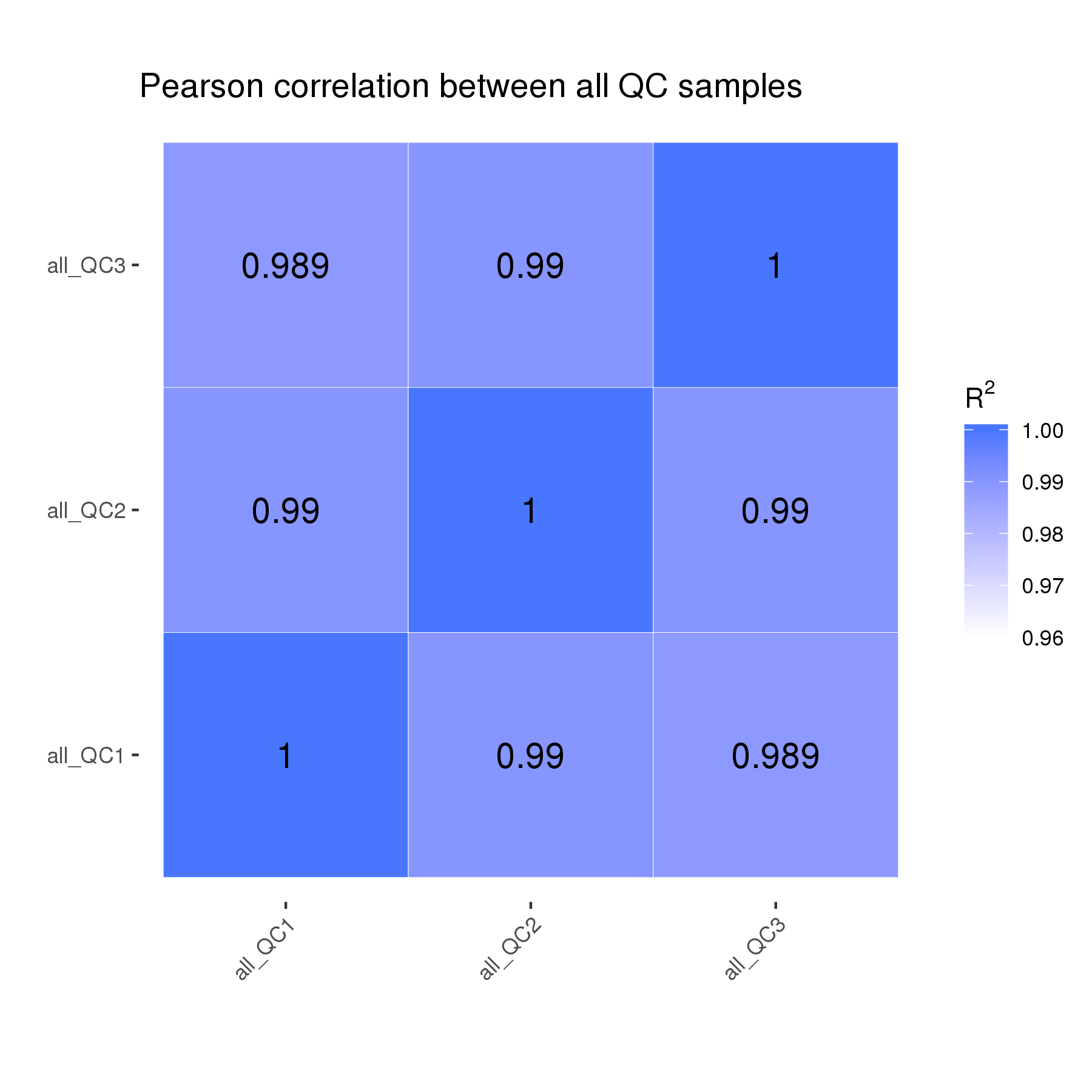

Supplement: Supplementary file 2 [file Image_1.PNG]

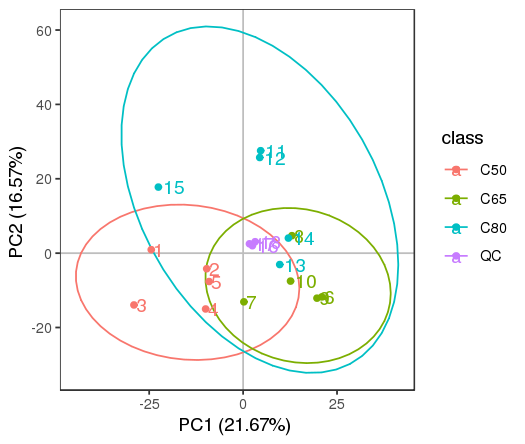

Supplement: Supplementary file 3 [file Image_2.PNG]
